# Obstructive Apnea and Hypopnea Length in Normal Children and Adolescents

**DOI:** 10.3390/brainsci11101343

**Published:** 2021-10-13

**Authors:** Lourdes M. DelRosso, David Panek, Greg Redding, Maria Paola Mogavero, Chris Ruth, Nicole Sheldon, Holly Blazier, Candace Strong, Maria Samson, Amy Fickenscher, Raffaele Ferri

**Affiliations:** 1 Division of Pulmonary and Sleep Medicine, Seattle Children’s Hospital, 4800 Sand Point Way, Seattle, WA 98105, USA; David.panek@ucsf.edu (D.P.); Gregory.redding@seattlechildrens.org (G.R.); chris.ruth@seattlechildrens.org (C.R.); Nicole.sheldon@seattlechildrens.org (N.S.); holly.blazier@seattlechildrens.org (H.B.); candace.strong@seattlechildrens.org (C.S.); maria.samson@seattlechildrens.org (M.S.); amy.fickenscher@seattlechildrens.org (A.F.); 2 Istituti Clinici Scientifici Maugeri, IRCCS, Scientific Institute of Pavia, Via Salvatore Maugeri 4, 27100 Pavia, Italy; paola_mogavero@libero.it; 3 Sleep Research Centre, Oasi Research Institute—IRCCS, Via C. Ruggero 73, 94018 Troina, Italy; rferri@oasi.en.it

**Keywords:** pediatric, obstructive sleep apnea, hypopnea length

## Abstract

(1) Background: Breathing is an essential function that requires both metabolic (or au-tomatic) and voluntary (behavioral) control during wakefulness but during sleep depends on metabolic control via peripheral and central chemoreceptors. Breathing during sleep disordered breathing also depends on the maturity of the neural centers and the strength of the respiratory muscles. We do not know if the response to apnea varies with age. (2) Methods: We measured the obstructive apneas and hypopneas during REM and NREM in polysomnography studies from children referred for snoring. Exclusion criteria: younger than 1 year of age, neuromuscular or syndrome comorbidity, oxygen or positive airway pressure, central apnea, and studies with loss of airflow sensors. (3) Results: Two-hundred-and-sixty-eight sleep studies were included. Mean age was 8.7 years (4.68 SD), range 1–18 years, 160 were male, and 108 were female. The 5th centile of apnea duration during NREM is above 8 s at all ages, with a tendency to increase in the oldest groups up to 10 s. During REM sleep, it shows a gradual increase from 6 s in the youngest children to 10 s in the oldest. (4) Conclusions: Apnea/hypopnea length increases with age in children and adolescents independently from sex or severity of OSA. Using adult criteria in teens seems to be accurate.

## 1. Introduction

Breathing is an essential function that requires both metabolic and behavioral control during wakefulness but depends on metabolic control during sleep. Breathing also depends on the maturity of the neural centers and the strength of the respiratory muscles. In patients with sleep disordered breathing, breathing after an apneic event results from the interaction between chemoreceptors relaying information to brainstem neurons responsible for generating breathing patterns and influencing respiratory motor neurons controlling the airway muscles; both of these mechanisms are also affected by sleep stage physiology [1]. With sleep onset, muscle tone and minute ventilation are reduced with a subsequent increase in upper airway resistance [2]. REM sleep further decreases tidal volume and protective airway reflexes [3]. In obstructive sleep apnea, the obstructive event termination usually occurs simultaneously with an arousal, triggered by hypoxia, hypercapnia, and the presence of an occluded airway [4]. Obstructive sleep apnea syndrome (OSAS) is a sleep breathing disorder characterized by repeated episodes of partial or complete upper airway obstruction during the night [5]. This obstruction usually manifests itself with a reduction (hypopnea) or complete cessation (apnea) of the air flow in the upper airways with intermittent hypoxia and an oxidative imbalance, with increased production of reactive oxygen species, tumor necrosis factors, cytokines inflammatory diseases (IL2, IL4, IL6), lipid peroxidation, and cell-free DNA [6]. An obstructive apnea or hypopnea typically ends when an upper airway obstruction triggers a reflex stimulation of respiratory effort by the diaphragm, intercostal, and abdominal muscles, which generates enough pressure to produce an arousal or a gasp, resulting in airway opening and post apnea hyperpnea [7]. Normal children with normal muscle strength should be able to generate this increased intrathoracic pressure in a somewhat predictable manner. The literature, however, lacks information regarding the amount of time that children require to terminate an obstructive event. There is also a lack of understanding about the relation of apnea length to various comorbidities, such as neuromuscular conditions. We also do not know if the response to apnea varies with age. The American Academy of Sleep Medicine (AASM) scoring manual [8] recommends scoring pediatric obstructive events based on a two-breath rule. To score a pediatric obstructive apnea, there must be a drop in respiratory signal excursion of at least 90% for at least the duration of two breaths during baseline breathing. To score a pediatric hypopnea, the drop in signal excursion needs to be at least 30% for the duration of two breaths. As respiratory rate decreases from birth to puberty, the duration of each breath becomes longer. In obstructive sleep apnea, there must be evidence of respiratory effort. The AASM scoring manual states that after 13 years of age, it is up to the discretion of the sleep physician to score the studies using the adult criteria, which differs from pediatric criteria. For adults, obstructive apneas or hypopneas must be scored if they last for a minimum of 10 s. The duration of obstructive sleep apneas and hypopneas as a continuum over the different ages of children and adolescents, however, has not been previously reported. 

In this study we analyzed obstructive apnea and hypopnea length in children undergoing polysomnography for the evaluation of snoring. We hypothesized that apnea/hypopnea length increases with age but should not be associated with the degree of obstructive sleep apnea. 

## 2. Materials and Methods

### 2.1. Subjects

**Figure 1:**
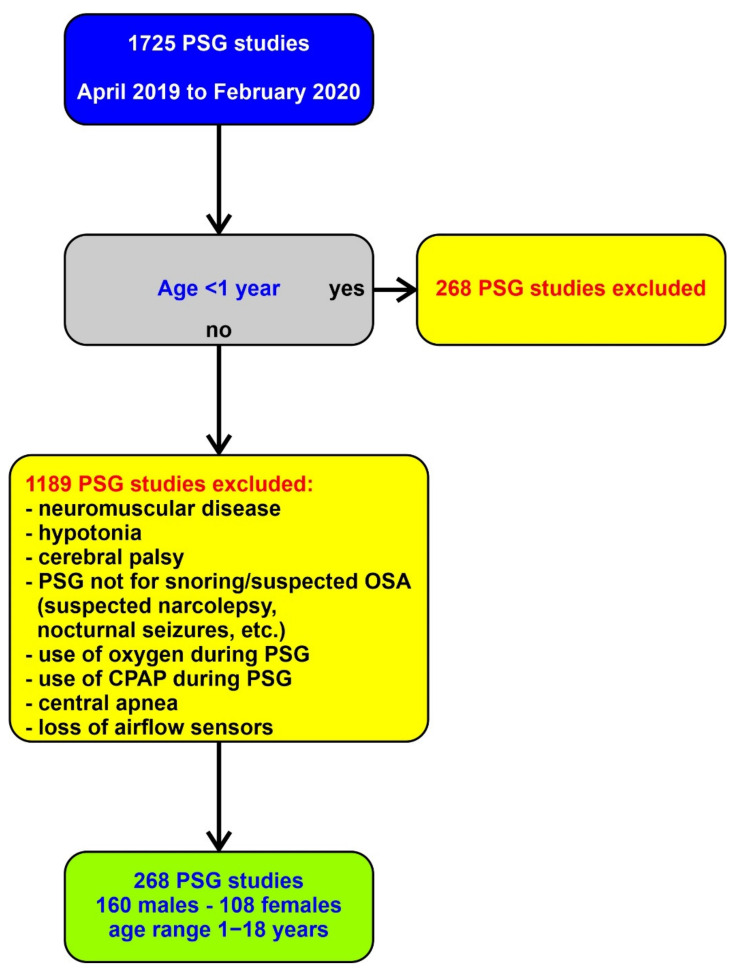
Shows the research protocol with inclusion and exclusion criteria.

We included polysomnography studies from children referred for the evaluation of snoring and suspected obstructive sleep apnea to the Sleep Disorders Center Seattle Children’s Hospital, Seattle, WA and who underwent polysomnography during the period between April 2019 and February 2020. All sleep studies were consecutively enrolled for analysis. We excluded studies from children younger than 1 year of age, because in our center, most studies in children younger than 1 year are performed secondary to laryngomalacia, hypoxemia, hypotonia, or genetic syndromes. We also excluded children older than 1 year, with neuromuscular disease, hypotonia, syndromes with hypotonia, cerebral palsy, children referred to a sleep study for non-respiratory reasons (e.g., suspected narcolepsy or nocturnal seizures), studies that used oxygen or positive airway pressure, children with central apnea, and studies with loss of airflow sensors. Figure 1 shows a summary of our protocol. 

The study was approved by the Seattle Children’s Hospital, Seattle, WA, Institutional Review Board Study 00002781.

### 2.2. Polysomnography

In-laboratory polysomnography (PSG) was performed according to the AASM criteria [9], and data were recorded using the Sandman Elite Natus system. Parameters recorded included electroencephalogram (EEG; two frontal, two central, and two occipital channels, referred to the contralateral mastoid); electro-oculogram, electromyogram (EMG) of the submentalis muscle, EMG of the right and left tibialis anterior muscles, respiratory signals, effort signals for thorax and abdomen, oximetry, capnography, a single-lead electrocardiogram, and video and audio recording. Calibrations were performed per routine standard by technicians. Epochs were scored by a certified sleep technologist and board-certified sleep physician according to the AASM criteria. Respiratory events were classified as obstructive apnea or hypopnea, according to the above-mentioned AASM criteria, and the duration and stage (NREM or REM) of each event were annotated by a certified sleep technologist. Obstructive sleep apnea (OSA) was defined as an obstructive apnea/hypopnea index (AHI) ≥ 1 events/hour [10].

### 2.3. Data, Analysis and Statistics Applied

The average values of all events of the same type were obtained for each subject enrolled, and these values were used for further analysis and for the calculation of the group mean values and their standard deviations as basic statistics.

We then checked for possible simultaneous effects of age, sex, and obstructive AHI (independent factors/predictors) on the duration of apnea and hypopnea during NREM and REM sleep separately (dependent variables) by means of the General Regression Models module offered by the commercially available software STATISTICA v.6, StatSoft Inc., Tulsa, OK, USA (this software was also used for all other statistical tests carried out in this study). This module allowed for the building of models for designs with categorical predictor variables, as well as with continuous predictor variables. For each dependent variable, three partial correlation coefficients were obtained, one for each independent factor, together with its statistical significance. Moreover, following the Cohen’s [11] indications, we considered correlation coefficients 0.10, 0.30, and 0.50 as corresponding to small, medium, and large sizes, respectively.

## 3. Results

In total, 268 pediatric polysomnograms were included in this study. The children’s mean age was 8.7 years (4.68 SD), the range was 1−18 years, 160 were male, and 108 were female.

Table 1 shows the results of the regression analysis taking into consideration the simultaneous effects of age, sex, and obstructive apnea/hypopnea index (the predictors) on both apnea and hypopnea during NREM and REM sleep (the dependent variables). Only age resulted to be significantly correlated with all four respiratory event durations, while both sex and severity of apnea did not seem to influence them. For this reason, our subsequent analyses focused on this correlation.

**Figure 2:**
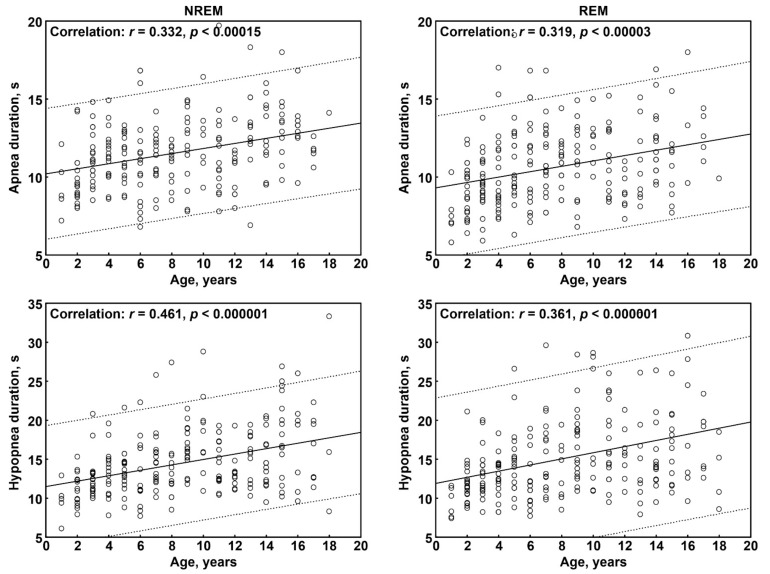
Correlation between age of participants and the respiratory event duration values considered in this study. The continuous line is the regression line, while the two dashed lines represent the limits of the area within which 95% of the points are expected.

Figure 2 shows, in detail, the correlation between age and each of the four respiratory event duration values. Again, all correlations were statistically significant, with duration showing a clear increase with age. All correlations were within the moderate-to-large size range (>0.30 to <0.50).

Mean duration values of apnea and hypopnea events during NREM and REM sleep, separately, for each age group are reported in Table 2, as well as the number of subjects from which each value was calculated.

**Figure 3:**
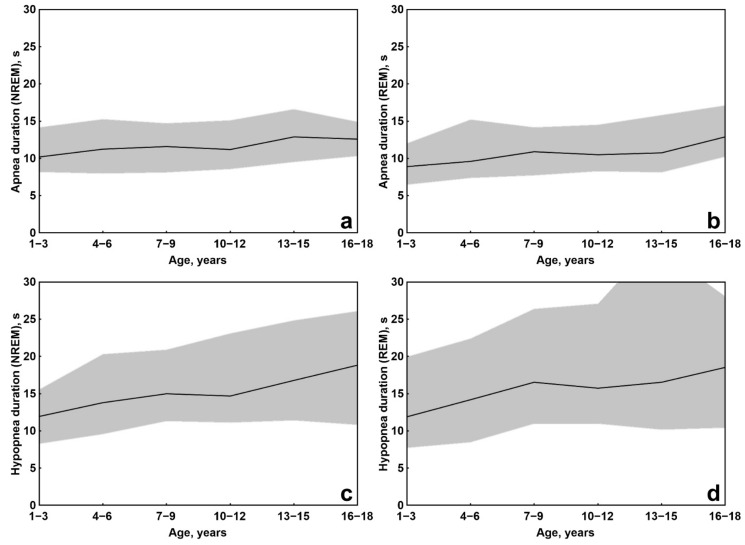
Median (continuous line) and the interval between the 5th and 95th centiles (grey-shaded area) of apnea duration during NREM (**a**) and REM sleep (**b**) and hypopnea duration during NREM (**c**) and REM sleep (**d**) in different age groups.

Finally, Figure 3 shows the median and the interval between the 5th and 95th centiles of apnea duration during NREM and REM sleep and hypopnea duration during NREM and REM sleep in different age groups (subjects were grouped into 3-year age subgroups: 1–3, 4–6, 7–9, 10–12, 13–15, and 16–18 years). This figure shows that the intersubject variability of apnea events is smaller than that of hypopnea events during both NREM and REM sleep; moreover, it tends to be constant at all ages, in contrast to that of hypopnea, which tends to be wider with advancing age, again, during both NREM and REM sleep. The graphs in Figure 3 also provide information on the minimum duration expected for the respiratory events, taking into consideration the 5th centile (bottom dotted line in each graph). The 5th centile of apnea duration during NREM sleep appears to be slightly above 8 s at all ages, with a slight tendency to increase in the oldest groups up to 10 s. During REM sleep, the 5th centile of apnea duration shows a gradual increase from approximately 6 s in the youngest children to 10 s in the oldest. The 5th centile of hypopnea duration during NREM sleep also shows a value just above 8 s in the youngest group and is longer than 10 s after the age of 6 years. Similarly, the 5th centile of hypopnea duration during REM sleep starts from values close to 8 s in the youngest group and is longer than 10 s after the age of 6 years.

## 4. Discussion

Our current study reports reference values for apnea and hypopnea length in children with sleep disordered breathing without any other comorbidities and demonstrates our original hypothesis that their duration is not correlated with severity of sleep disordered breathing but with age. We have also demonstrated that the mean apnea/hypopnea length of 10 s is usually achieved at an earlier age than adolescence, but its minimum duration can be less than 10 s. If we consider that, at ages below 15 years, the respiratory rate is >20 breaths/min, the duration of two respiratory cycles is <6 s. Our data-driven analysis shows that an appropriate lower limit for apnea/hypopnea events might be 7−8 s in the youngest children and 10 s above the age of 6 years (as in adults). 

There are currently no studies reporting apnea/hypopnea length in school age children and adolescents or across the continuum of the lifespan. Brockmann et al. [12] reported the median duration of obstructive apneas in infants at 1 month of age to be 5.1 s and the duration of hypopneas to be 6.6 s. In our study, mean apnea/hypopnea length at 1 year of age ranged from 7.1−10.7 s, demonstrating a longer duration of events than in infants. Also, in their study, Brockmann et al. [12] demonstrated that obstructive apnea was not as predominant in infants, with a mean obstructive AHI of 0.8/hour, while central apneas were more prominent in this age group, with a central apnea index of 4.1/hour. Of note, the duration of central events matched that of obstructive apneas, lasting 5.1 s [12]. Some studies in adults have measured apnea lengths at various ages. For example, McBrayer et al. [13] also found that apneas increased in length with age in adults but were longer in men than in women. For instance, in men, the duration of events during NREM sleep increased from 20.1 s in men aged 18−39 years to 23.8 s in the older age group (60-88 years), while in women in the younger age group (18−39 years), the apnea duration was 16.7 s compared to 20.6 s in the older age group [13]. During REM sleep, the events were longer than during NREM and also increased in duration with age. The authors also postulated that upper airway muscle tone can be responsible for the changes in apnea duration seen with aging [13]. These results match our findings, but we did not find a sex difference in children. 

The prolongation of the duration of obstructive apneic events associated with age can be postulated to occur secondary to various mechanisms: upper airway muscle tone changes [14], reflex changes in chemoreceptor sensitivity [15], and shifts in sleep stages that occur naturally with age [16]. 

Benefits from studying apnea length in various patient populations have been previously published. For instance, Efken et al. [17] found longer apneas (20.5 s vs. 16.5 s) in a subset of 13 adult patients with heart failure and OSA in comparison to 26 control patients with OSA but without heart failure, pointing towards a different phenotype of OSA apnea/hypopnea in patients with heart failure. Wu et al. [18] analyzed polysomnographic data of 596 adults with OSA (AHI ≥ 5/hour) and found that higher mean apnea/hypopnea duration was associated with higher odds of moderate-to-severe systemic hypertension. There are currently no studies assessing apnea or hypopnea length in children with neuromuscular abnormalities, for example. We believe our data in children without comorbidities will serve as a reference to be able to compare obstructive events to those in children with neurologic or neuromuscular conditions and assess if the muscular weakness contributes to difficulty in the generation of pressure enough to open the airway. There are numerous studies that have identified various contributors to OSA in children and in adults that vary according to the age of the patient; in younger children, for instance, adenotonsillar hypertrophy plays a role, while in adolescents and adults other factors can be more contributory. Equally, OSA is considered an inflammatory systemic disease, and various degrees of inflammation could potentially contribute to apnea length, although in our study the severity of disease was not correlated with the length of the apnea. The contribution of these anatomical factors to the apnea length are potential areas of further research.

The limitations of our study include a single center experience with a relatively small number of children without significant comorbidities, and sample size not being calculated. It should be acknowledged that the low number of subjects in some age category (especially the older subjects aged 16–18 years) does not allow us to draw firmer conclusions about them from this analysis.

## 5. Conclusions

In conclusion, we have found that apnea and hypopnea length increases with age in children and adolescents without association with sex or the severity of OSA, and we have provided reference values in children without neuromuscular or neurologic conditions. 

## Figures and Tables

**Table 1:** Analysis of the simultaneous effects of age, sex, and obstructive apnea/hypopnea index on the duration of apnea and hypopnea during NREM and REM sleep, separately. The partial correlation coefficients are reported for each predictor, as well as their statistical significance.

		Age	oAHI	Sex
Apnea, NREM	Correlation	0.287	−0.015	0.067
	F	13.410	0.036	0.664
	p<	0.00035	NS	NS
Apnea, REM	Correlation	0.304	−0.032	−0.035
	F	15.168	0.155	0.187
	p<	0.00015	NS	NS
Hypopnea, NREM	Correlation	0.442	0.007	0.003
	F	36.165	0.007	0.001
	p<	0.000001	NS	NS
Hypopnea, REM	Correlation	0.390	−0.035	−0.119
	F	26.710	0.188	2.122
	p<	0.000001	NS	NS
oAHI = obstructive apnea/hypopnea index; NS = not significant.				

**Table 2:** Mean duration of apnea and hypopnea events, during NREM and REM sleep, separately, for each age group.

	Apnea, NREM		Apnea, REM		Hypopnea, NREM		Hypopnea, REM	
Age, Years	N	Mean ± SD	N	Mean ± SD	N	Mean ± SD	N	Mean ± SD
1	5	9.4 ± 1.9	6	7.1 ± 0.7	6	10.7 ± 2.8	6	9.2 ± 1.8
2	19	9.8 ± 1.8	19	9.2 ± 1.7	18	11.3 ± 2.3	19	12.5 ± 3.8
3	20	11.3 ± 1.6	19	9.3 ± 1.7	20	12.8 ± 2.7	19	13.6 ± 3.2
4	17	11.4 ± 1.7	17	10.0 ± 2.8	18	13.1 ± 3.2	18	12.7 ± 3.2
5	19	11.4 ± 1.4	17	10.8 ± 2.8	18	14.1 ± 2.6	17	15.5 ± 4.5
6	18	11.1 ± 3.1	13	11.1 ± 2.7	18	15.0 ± 4.3	16	15.4 ± 6.7
7	18	11.3 ± 1.6	17	10.9 ± 2.4	20	14.9 ± 3.7	17	16.4 ± 4.9
8	12	10.7 ± 1.2	10	11.3 ± 1.6	12	14.5 ± 4.3	8	16.0 ± 3.7
9	15	12.2 ± 2.5	14	10.7 ± 2.2	18	16.3 ± 2.9	18	17.8 ± 5.1
10	7	12.5 ± 2.3	6	12.6 ± 1.6	9	18.8 ± 4.8	8	19.0 ± 7.4
11	16	11.7 ± 2.8	12	11.2 ± 2.3	19	15.4 ± 3.5	17	17.1 ± 4.8
12	10	10.5 ± 1.7	9	9.1 ± 1.2	12	13.5 ± 2.6	9	15.7 ± 3.1
13	11	13.0 ± 2.8	7	10.1 ± 2.1	13	16.1 ± 3.6	11	18.0 ± 9.6
14	13	12.4 ± 2.2	10	12.4 ± 2.4	16	16.2 ± 3.6	12	16.1 ± 4.4
15	11	13.3 ± 2.2	7	10.5 ± 2.8	17	19.4 ± 4.8	15	22.6 ± 11.9
16	9	13.2 ± 1.9	1	18.0	9	20.2 ± 5.4	6	21.2 ± 7.5
17	5	11.6 ± 0.7	4	12.8 ± 1.6	8	16.8 ± 4.0	7	18.4 ± 3.4
18	1	14.1	1	9.9	4	18.9 ± 10.5	4	13.3 ± 4.4

## Data Availability

The data presented in this study are available on request from the corresponding author. The data are not publicly available due to privacy protection reasons.
